# Molecular Pathways Associated With Methylmercury-Induced Nrf2 Modulation

**DOI:** 10.3389/fgene.2018.00373

**Published:** 2018-09-12

**Authors:** Takamitsu Unoki, Masahiro Akiyama, Yoshito Kumagai, Filipe Marques Gonçalves, Marcelo Farina, João Batista Teixeira da Rocha, Michael Aschner

**Affiliations:** ^1^Department of Basic Medical Sciences, National Institute for Minamata Diseasexy3Minamata, Japan; ^2^Environmental Biology Laboratory, Faculty of Medicine, University of Tsukuba, Tsukuba, Japan; ^3^Department of Molecular Pharmacology, Albert Einstein College of Medicine, Bronx, NY, United States; ^4^Department of Biochemistry, Federal University of Santa Catarina, Florianópolis, Brazil; ^5^Department of Biochemistry, Federal University of Santa Maria, Santa Maria, Brazil

**Keywords:** methylmercury, toxicity, Nrf2, gene expression, central nervous system

## Abstract

Methylmercury (MeHg) is a potent neurotoxin that affects particularly the developing brain. Since MeHg is a potent electrophilic agent, a wide range of intracellular effects occur in response to its exposure. Yet, the molecular mechanisms associated with MeHg-induced cell toxicity have yet to be fully understood. Activation of cell defense mechanisms in response to metal exposure, including the up-regulation of Nrf2- (nuclear factor erythroid 2-related factor 2)-related genes has been previously shown. Nrf2 is a key regulator of cellular defenses against oxidative, electrophilic and environmental stress, regulating the expression of antioxidant proteins, phase-II xenobiotic detoxifying enzymes as well phase-III xenobiotic transporters. Analogous to other electrophiles, MeHg activates Nrf2 through modification of its repressor Keap1 (Kelch-like ECH-associated protein 1). However, recent findings have also revealed that Keap1-independent signal pathways might contribute to MeHg-induced Nrf2 activation and cytoprotective responses against MeHg exposure. These include, Akt phosphorylation (Akt/GSK-3β/Fyn-mediated Nrf2 activation pathway), activation of the PTEN/Akt/CREB pathway and MAPK-induced autophagy and p62 expression. In this review, we summarize the state-of-the-art knowledge regarding Nrf2 up-regulation in response to MeHg exposure, highlighting the modulation of signaling pathways related to Nrf2 activation. The study of these mechanisms is important in evaluating MeHg toxicity in humans, and can contribute to the identification of the molecular mechanisms associated with MeHg exposure.

## Introduction

Methylmercury (MeHg) is a potent neurotoxin that affects particularly the developing brain and its exposure remains a public health concern. The metal in its inorganic form (Hg^0^, Hg^+^, or Hg^2+^) is present in the environment due to both natural and anthropogenic sources. Aquatic microorganisms are capable of converting inorganic mercury to MeHg, which accumulates in the food chain, reaching maximum concentrations in long-lived and predatory fish, such as swordfish or tuna (Clarkson et al., 2003; Farina et al., 2011b; Ceccatelli et al., 2013). Therefore, the consumption of fish and seafood are the main source of MeHg in humans, and greater than 90% of this toxicant is absorbed by the gastrointestinal tract and it is ubiquitously distributed. MeHg can easily cross the blood–brain barrier (BBB) via the neutral amino acid transport system l as a complex with L-cysteine, and its concentrations can reach 3–6 fold higher levels in the central nervous system (CNS) than in the blood (Costa et al., 2004; Johansson et al., 2007; Yin et al., 2008; Grandjean and Herz, 2011; Syversen and Kaur, 2012).

The molecular mechanisms associated to MeHg-induced cell toxicity have yet to be fully understood. MeHg accumulates in numerous cell types in the CNS, but predominantly in astrocytes (Charleston et al., 1994, 1996; Aschner, 1996). MeHg impairs glutamate and aspartate uptake in astrocytes, leading to increased glutamate concentration in the synaptic cleft, which in turn, induces neuronal cell death secondary to excitotoxicity (Aschner et al., 2007). MeHg has high affinity for thiol and selenol groups and its interaction with these groups in amino acids residues may alter the structure of a large number of proteins, leading to mitochondrial dysfunction, decreased glutathione levels, disruption of calcium homeostasis and an increase in reactive oxygen species (ROS) production (Limke et al., 2004; Falluel-Morel et al., 2007; Ceccatelli et al., 2010; Farina et al., 2011a).

A substantial body of research has focused on the role of increased ROS production and impairment of antioxidant cellular defense in MeHg-induced cell toxicity. Several studies have demonstrated that the activation of the antioxidant system can afford protection against MeHg toxicity (Do Nascimento et al., 2008; Hwang, 2012; Feng et al., 2017). Activation of proteasome–ubiquitination system, autophagy, heat shock factor protein 1 (Hsf1) and increased cellular metabolites, such as hydrogen sulfide, have all been associated with cell defense responses in the face of MeHg-induced cell toxicity (Hwang et al., 2002, 2011; Yoshida et al., 2011). In this sense, the study of these mechanisms is critical in evaluating MeHg toxicity and can contribute to the identification of the molecular mechanisms associated to MeHg exposure.

Nrf2 (nuclear factor erythroid 2-related factor 2) is a key regulator in cellular defenses against oxidative, electrophilic and environmental stress. In the nucleus, up-regulation of the transcription factor Nrf2 in response to MeHg exposure has been noted (Toyama et al., 2007; Kumagai et al., 2013). In this review, we summarize the most recent findings associated to Nrf2 up-regulation in response to MeHg exposure and also the modulation of other signaling pathways related to Nrf2 activation that might also be associated with cell defense responses against MeHg toxicity.

## Nrf2 Activation Mechanisms

Once activated, Nrf2 is translocated into the cell nucleus and forms a dimer with small Maf protein (sMaf) and binds to antioxidant/electrophile response elements (AREs/EpREs) located in the regulatory regions of many responsible genes for cellular defense (Itoh et al., 1997). Nrf2 cooperatively regulates antioxidant proteins such as glutamate cysteine ligase (GCL) and heme oxygenase-1 (HO-1), phase-II xenobiotic detoxifying enzymes, and phase-III xenobiotic transporters such as multidrug resistance-associated proteins (MRPs) (Itoh et al., 1997; Alam et al., 1999; Wild et al., 1999; Chan and Kwong, 2000; Hayashi et al., 2003; Vollrath et al., 2006; Maher et al., 2007).

Nrf2 is regulated by Kelch-like ECH-associated protein 1 (Keap1), an adaptor subunit of Cullin 3-based E3 ubiquitin ligase. Under normal conditions, Keap1 binds to Nrf2 in the cytoplasm and promotes the ubiquitination and proteasomal degradation of Nrf2, and thus, acts as a negative regulator of Nrf2 (Itoh et al., 1999; Ishii et al., 2000; Wakabayashi et al., 2003). Keap1 acts as a sensor protein for oxidative and electrophilic insults through the modification of its highly reactive cysteine residues (e.g., Cys151, Cys273, and Cys288) (Dinkova-Kostova et al., 2002; Zhang and Hannink, 2003; Eggler et al., 2005; Hong et al., 2005). Thus, when the interaction between Nrf2 and Keap1 is disrupted, proteasomal degradation of Nrf2 decreases, causing *de novo* Nrf2 to build up within the cell, leading to increased translocation of Nrf2 into the nucleus.

In addition to repression of Nrf2 activity by Keap1, glycogen synthase kinase-3 (GSK-3) has been shown to phosphorylate specific serine residues in the Neh6 domain of Nrf2, creating a degradation domain that is recognized by the ubiquitin ligase adapter β-transducin repeat-containing protein (β-TrCP) and subsequently targeted for proteasomal degradation independent of Keap1 (Rada et al., 2011, 2012; Chowdhry et al., 2013; Cuadrado, 2015).

## Cytoprotective Responses to MeHg

### MeHg Regulation on Nrf2 Activity

Since MeHg is a potent electrophilic agent, a wide range of intracellular effects is associated with exposure to it (Farina et al., 2011a,b). Among its effects, MeHg is able to modulate Keap1/Nrf2 signaling. Data from *in vivo* experiments have shown increased gene expression and proteins related to Nrf2 activation, such as HO-1 (heme-oxigenase-1) and γ- GCS (γ-Glutamylcysteine synthetase) (Feng et al., 2017). It has been shown that MeHg can induce the transcription of several genes related to Nrf2 activation including HO-1, NQO-1 [NQO1 NAD(P)H quinone dehydrogenase], GCLC (glutamate-cysteine ligase catalytic subunit) and Nrf2 in several cells types such as SH-SY5Y neuroblastoma cells, primary hepatocytes, microglia and astrocytes, to name a few (Toyama et al., 2007; Wang et al., 2009; Ni et al., 2011; Culbreth et al., 2017).

Once Nrf2 orchestrates the transcription of antioxidant-related genes and phase-2 detoxification enzymes, their by products afford neuroprotection. For example, Toyama et al. (2007) have demonstrated that primary hepatocytes derived from Nrf2^(−/−)^ mice showed greater susceptibility to MeHg-induced cell death than cells derived from wild-type animals. It was also observed that Nrf2^(−/−)^ animals exposed to MeHg for 8 days (5 mg/kg/day) exhibited flaccidity in the posterior limbs, but this effect was absent in wild-type mice exposed to MeHg. Furthermore, 3 weeks after the first administration of MeHg, all of the Nrf2^(−/−)^ animals died as a result of MeHg-toxicity while all the wild-type mice survived an analogous exposure protocol (Toyama et al., 2011). In agreement with this observation, it has been also demonstrated that Nrf2 knockdown augments the *in vitro* toxic effect of MeHg in primary microglia and astrocytes cultures, as well as in SH-SY5Y cells (Toyama et al., 2007; Ni et al., 2011). Moreover, it has been reported that several natural compounds like flavonoids and isothiocyanates can up-regulate Nrf2 activity and afford neuroprotection against MeHg. *In vitro* treatment with tea polyphenols in astrocytes increased the expression of genes downstream of Nrf2 activity (such as Nrf2, HO-1, and GCSH), preventing the MeHg-induced decrease in cell viability, glutathione (GSH) content, and the increase in ROS production (Liu et al., 2016). Feng et al. (2017) have recently shown that treatment with sulforaphane, a natural dietary constituent that induces Nrf2 (Sun et al., 2017), prevented some of the *in vivo* MeHg toxic effects in mice cerebral cortex and also reinforced the MeHg-induced Nrf2 up-regulation (Feng et al., 2017). In primary hepatocytes, the up-regulation of Nrf2 was also associated to an increase in antioxidant responses, raising GSH levels, and increased MeHg export from the cell (Toyama et al., 2011). Taken together, these data reinforce the hypotheses that Keap1/Nrf2 pathway may exerts protection against MeHg toxicity.

### Keap1-Dependent Nrf2 Regulation by Methylmercury

**FIGURE 1:**
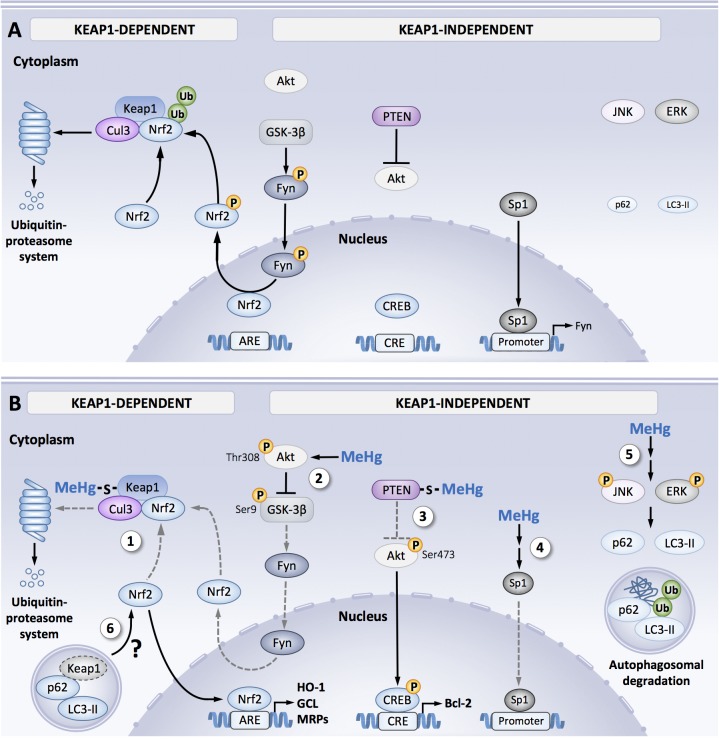
Signal orchestration against MeHg toxicity. **(A)** Under non-stressed conditions, Nrf2 is captured by Keap1 and ubiquitinated by Cul3 in the cytosol that leads to degradation through ubiquitin–proteasome system, resulting in the inhibition of Nrf2 translocation from the cytoplasm to nucleus. Fyn is phosphorylated by GSK-3β, leading to Fyn nuclear localization. Fyn phosphorylates nuclear Nrf2, which leads to nuclear export and degradation of Nrf2. Sp1 is a transcription factor of Fyn. Steady state level of p62 and LC3-II expression under basal activity of JNK and ERK. **(B)** Cellular protective responses to MeHg. MeHg covalently modifies Keap1 through Cys151 and/or Cys319, leading to inhibition of Nrf2 degradation. As a result, Nrf2 translocates into nucleus and the interacts with a partner protein sMaf, resulting in formation of heterodimer that binds to the antioxidant response element (ARE), thereby upregulating its downstream genes (e.g., HO-1, GCL, and MRP) (1). MeHg induces phosphorylation of GSK-3β at Ser9 mediated by activated Akt at Thr308. Since this inactive form of GSK-3β is unable to phosphorylate Fyn, substantial retain of nuclear Nrf2 coupled to diminished nuclear translocation of Fyn occurs (2). MeHg covalently modifies PTEN, resulting in inhibition of its catalytic activity, thereby phosphorylating Akt through Thr473 and tits downstream transcription factor CREB, which binds to the cAMP-response element (CRE), leading to up-regulation of anti-apoptotic protein Bcl-2 (3). MeHg reduces Sp1 protein level and thus down-regulates Sp1-dependent target genes such as *fyn* (4). Phosphorylation of JNK and ERK mediated by MeHg increases p62 and LC3-II expressions, thereby promotes autophagosomal degradation of misfolded/damaged proteins (5). Possible linkage between MeHg-induced MAPK activation and Nrf2 upregulation via p62/LC3-II-mediated autophagosomal degradation of Keap1 (6). Dotted gray lines indicate processes disrupted by MeHg exposure.

The molecular mechanisms associated to MeHg-induced Nrf2 activation are complex and have yet to be fully understood. **Figure 1** shows the principle findings related to MeHg-induced regulation of Keap-1/Nrf2. Keap-1 is the main negative regulator of Nrf2 activity and contains 27 (in the human isoform) and 25 (in the mouse isoform) cysteine residues that can act as a potential target to MeHg (Dinkova-Kostova et al., 2002; Zhang and Hannink, 2003). MeHg binds to recombinant Keap1 protein and results from Biotin-PEAC_5_-maleimide-labeling assay revealed that Keap-1 is a target for *S*-mercuration in SH-SY5Y cells (Toyama et al., 2007, 2013). Additional studies with MALDI-TOF/MS have indicated that MeHg can bind specifically to three cysteines residues in Keap1 structure: Cys151, Cys368, and Cys489 (Kumagai et al., 2013). Among these three residues, Cys151 is essential to electrophile-mediated disassociation of Keap1 and Nrf2, and also Keap1-directed ubiquitination (Levonen et al., 2004). Thus, one might speculate that MeHg-induced modifications in the Cys151 residue are associated with changes in the Keap1/Nrf2 signaling induced by MeHg. Within the intracellular space, MeHg is readily conjugated to GSH forming MeHg-SG adduct that is transferred to extracellular space by the MRP (Farina et al., 2011a). Taking advantage of a synthetic ethyl monoester of MeHg-SG, Yoshida et al. (2014) have shown that this adduct effectively induced concentration-dependent toxicity and activated Nrf2-related genes in SH-SY5Y cells. The Hg-S bond in MeHg-SG adducts is relatively unstable, thus GSH adducts readily undergo *S*-transmercuration with cellular proteins including Keap1, forming protein-MeHg adducts. While MeHg can modify the Cys151 residue in the Keap1 structure, the MeHg-SG adduct was able to modify the Cys319 residue. This residue is located in the intervening region of the Keap1 structure and is important for the ubiquitin E3 ligase activity and Nrf2 degradation (Taguchi et al., 2011). Thus, it is possible to speculate that the *S*-mercuration in the Cys319 residue also contributes to the up-regulation of Nrf2 in response to MeHg (Toyama et al., 2013). Ultimately, it is noteworthy that ROS are also important regulators of Nrf2 activation, thus representing one of the main mechanisms that mediate MeHg toxicity (Farina et al., 2011a; Kumagai et al., 2013; Tebay et al., 2015). However, further studies are necessary to elucidate the role of ROS in Nrf2 activation by MeHg.

### Keap1-Independent Nrf2 Regulation by Methylmercury

Culbreth et al. (2017) found a Keap1-independent Nrf2 activation pathway in MeHg exposed rat primary astrocytes, possibly involving the regulation of a member of the src family kinase named Fyn. It has been reported that Fyn can phosphorylate Nrf2 favoring its exports from the cell nucleus (Jain and Jaiswal, 2007; Niture et al., 2014). MeHg exposure *in vitro* increased gene expression of HO-1 and NQO-1 as well as nuclear Nrf2 localization. In parallel to increased Nrf2 activity, MeHg also induced a decrease in Fyn mRNA expression and protein nuclear localization, suggesting a Fyn downregulation in astrocytes (**Figure 1**).

In primary cultures of rat astrocytes, the downregulation of Fyn expression was also followed by a decrease in Sp1 protein levels, the transcription factor that regulates Fyn expression. Noteworthy, MeHg can also decrease the expression of other genes related to Sp1 activation such as TGF-β1 (Culbreth et al., 2017) (**Figure 1**). This result is in line with earlier findings showing Sp1 interaction with Nrf2 at promoter sequences that represses Sp1-specific target gene expression (Gao et al., 2014). In contrast, MeHg exposure SH-SY5Y cells has been shown to induce Sp1 activation, which was associated with increased p38^MAPK^ phosphorylation and HDAC4 expression. Furthermore, the knockdown of p38^MAPK^, Sp1, and HDAC4 afforded neuroprotection against MeHg toxicity in cortical primary neurons (Guida et al., 2017). Thus, it seems that a decrease in Sp1 function was associated with a cytoprotective response in astrocytes (Nrf2 activation), but the activation of this transcription factor might be associated with neuronal cell death. Further studies are necessary to dissect out the role of Sp1 in MeHg toxicity.

As shown in **Figure 1**, MeHg also induced Akt phosphorylation (Thr308) (Culbreth et al., 2017) confirming previous results showing the knockdown of the p85α regulatory subunit of phosphoinositide 3-kinase (PI3K) attenuated MeHg-induced Nrf2 activation (Wang et al., 2009). This finding corroborates previous reports that have shown a correlation between Akt activation and up-regulation of Nrf2-related gene expression (Calkins et al., 2009; De Oliveira et al., 2015; Wang et al., 2016). Once activated, Akt promotes inhibitory phosphorylation of GSK-3β (Ser9), inhibiting Fyn phosphorylation and nuclear export (Jain and Jaiswal, 2007; Culbreth et al., 2017). Furthermore, GSK-3β activation is associated with increased Nrf2 phosphorylation associated with its degradation (Rada et al., 2011; Niture and Jaiswal, 2012). Since there is no evidence for a direct Nrf2 phosphorylation by Akt, it is reasonable speculate that the modulation of GSK-3β and/or Fyn afford a link between MeHg-induced activation of Akt and Nrf2 (**Figure 1**).

### Modulation of Akt, MAPK, and Autophagy in Response to MeHg Exposure

In addition to Nrf2 activation, Akt up-regulation can modulate other signaling pathways related to cell survival, and exert cytoprotective effects against MeHg toxicity. Impairment in Akt signaling has been shown to exacerbate MeHg-induced cell death (Wang et al., 2009; Unoki et al., 2016). Exposure of SH-SY5Y cells to MeHg increased Akt phosphorylation and nuclear localization, CREB phosphorylation and Bcl-2 protein levels (Unoki et al., 2016), as shown in **Figure 1**. These results corroborate earlier findings that showing increased CREB phosphorylation in the cerebral cortex of rats exposed to MeHg (Fujimura and Usuki, 2017). Akt phosphorylation is regulated, to a certain extent, by the phosphatase activity of PTEN (phosphatase and tensin homolog deleted on chromosome 10). Notably, MeHg (1 μM) decreased PTEN activity *in vitro* probably due to *S*-mercuration and/or by ROS-induced modifications in cysteine residues in the enzyme structure (Unoki et al., 2016). However, at higher concentrations (>10 μM), MeHg disrupted this signaling pathway. Although the PTEN *S*-mercuration persisted after exposure to higher MeHg concentrations, decreased Akt phosphorylation and CREB activity were noted, likely due to increased *S*-mercuration of these proteins (Unoki et al., 2016). Thus, one may posit that some of the effects associated with MeHg are concentration- or cell type-dependent, and could be related to a cytoprotective or toxic effect (Unoki et al., 2016). This hypothesis is also consistent with reports that have shown down-regulation of Akt in response to MeHg, such as decreased Akt phosphorylation in the hippocampus of 30-day-old rat pups prenatally exposed to MeHg (Heimfarth et al., 2018a). MeHg also reduced Akt phosphorylation *in vitro* in primary neurons, at concentrations associated with cell death (Pierozan et al., 2017). Nonetheless, further studies are necessary to elucidate the biochemical mechanisms associated to the regulation of Akt signaling induced by MeHg.

In addition to Akt activation, other signaling pathways have been shown to be activated by MeHg. A role for mitogen activated protein kinase (MAPK) has been advanced in the cellular responses to MeHg. MAPKs are a group of serine–threonine kinases associated with a various range of cell processes, such as inflammation, cellular survival, differentiation, or death (Cargnello and Roux, 2011; Kim and Choi, 2015). MeHg has been shown to activate members of the MAPK kinase family, especially the extracellular signal-regulated kinases 1/2 (ERK1/2) (Sakaue et al., 2009; Lu et al., 2011), c-Jun N-terminal kinases (JNKs) (Fujimura et al., 2009), and p38^MAPK^ (Guida et al., 2017; Heimfarth et al., 2018a). MeHg has also been reported to down-regulate MAPK. A decrease in ERK 1/2 and JNK 1/2 phosphorylation in young rats in response to prenatal exposure to MeHg has been noted (Heimfarth et al., 2018a,b). NGF-induced ERK1/2 phosphorylation in PC12 cells was also attenuated by MeHg (Parran et al., 2004; Fujimura and Usuki, 2015). Therefore, it remains unclear whether MAPK activation is consistently associated with MeHg-induced cell death, and the possibility that the activation of these signaling pathways is associated with cell defense mechanisms may not be excluded.

Takanezawa et al. (2016) have demonstrated that MeHg can increase ERK 1/2, JNK, and p38^MAPK^ phosphorylation, which might be related to the induction of the autophagy process, playing a protective role against MeHg toxicity (**Figure 1**). In eukaryotic cells, autophagy is an important pathway responsible for the degradation/elimination of misfolded and damaged proteins, non-functional organelles and protein aggregates (Mizushima, 2007; Twayana and Ravanan, 2018). Autophagy regulation is a complex process that involves the regulation of other signaling pathways besides MAPK activation, such as the modulation of kinases such as: PI3K, mammalian target of rapamycin (mTOR) and adenosine-monophosphate-activated protein kinase (AMPK). It has been suggested that the autophagy regulation by MAPK is an indirect process, that involves the regulation of some of autophagy components expression (Sridharan et al., 2011). ERK and JNK activation could be associated to p62 induction, and also increases Beclin-1 expression that promotes LC3-1 to LC3-II conversion, which serves as an indicator of the autophagy process progression (Mizushima, 2007; Sridharan et al., 2011; Zhou et al., 2015; Twayana and Ravanan, 2018). Corroborating these observations, pharmacological inhibition of JNK and ERK activation attenuated the MeHg-induced increase in p62 and LC3-II levels (Takanezawa et al., 2016) (**Figure 1**). Moreover, it is noteworthy that the p62 role in cell function goes beyond the autophagy regulation. p62 could interact with ubiquitinated proteins favoring its autophagosomal degradation. However, it has been reported that the KIR domain in p62 can interacts with the Kelch domain in Keap1 structure inducing its degradation and consequent Nrf2 stabilaztion and increase in gene expression (Silva-Islas and Maldonado, 2018). Thus, it is reasonable to speculate that MeHg regulates Nrf2 activity through p62 modulation, illustrating a crosstalk between Nrf2 modulation and MeHg-induced autophagy, leading to cell survival against metal-toxicity (**Figure 1**).

## Conclusion

Many reports have set Keap1/Nrf2 pathway to dogma in antioxidant/electrophile response. Like other electrophiles, MeHg also activates Nrf2 through modification of Keap1 and might induces p62-mediated autophagosomal degradation of Keap1. However, as mentioned before, recent findings have revealed that Keap1-independent signal pathways also contribute to the cytoprotective response against MeHg exposure as Akt phosphorylation (Akt/GSK-3β/Fyn-mediated Nrf2 activation pathway) and PTEN/Akt/CREB pathway. However, it remains unclear if all of these mechanisms are inherent to all cell types exposed to MeHg or are cell-specific. In addition to Keap1/Nrf2 pathway, Akt activation may sustain and expand cytoprotective response against MeHg toxicity through nuclear Nrf2 stabilization and CRE dependent (but not ARE dependent) target genes upregulation. It has been reported that the Nrf2 pathway can regulate Bcl-2 to block apoptosis (Niture and Jaiswal, 2012), which may suggest a cross talk between these pathways (Chuang et al., 2015; Mylroie et al., 2015). In this regard, further studies would be necessary to elucidate if these molecular events can occur in the context of MeHg exposure. Specific modulation of these pathways may afford targets for the development of new therapeutic strategies for the treatment of MeHg intoxication in humans. Taken together, the data discussed in this paper corroborate the pivotal role of Nrf2 up-regulation as a molecular mechanism in combatting MeHg-induced toxicity and further alluding to novel directions for future research in identifying molecular mechanisms associated to MeHg exposure.

## Author Contributions

All authors listed have made a substantial, direct and intellectual contribution to the work, and approved it for publication.

## Conflict of Interest Statement

The authors declare that the research was conducted in the absence of any commercial or financial relationships that could be construed as a potential conflict of interest. The reviewer KB and handling Editor declared their shared affiliation.
